# Comparison of thyroid function in different emotional states of drug-naïve patients with bipolar disorder

**DOI:** 10.1186/s12902-021-00869-5

**Published:** 2021-10-21

**Authors:** Shengnan Zhao, Xu Zhang, Yaling Zhou, Hao Xu, Yuwei Li, Yuexin Chen, Bo Zhang, Xueli Sun

**Affiliations:** 1 Mental Health Center, West China Hospital, Sichuan University, Chengdu, Sichuan China; 2 Sichuan Provincial Center for Mental Health, Sichuan Academy of Medical Sciences & Sichuan Provincial People’s Hospital, Chengdu, Sichuan China

**Keywords:** Bipolar disorder, Bipolar depression, Bipolar mania, Thyroid function

## Abstract

**Background:**

Previous studies have shown that bipolar disorder is closely related to thyroid dysfunction. Psychiatric drugs have a large or small effect on thyroid function, and thyroid hormone levels can also affect the effect of drug treatment. Therefore, the purpose of this study is assessment the thyroid function of drug-naive bipolar disorder across different mood states, with the expectation of providing support for treatment options.

**Methods:**

The present study is a cross-sectional study. Patients diagnosed with bipolar disorder according to the International Classification of Diseases diagnostic Criteria, Edition 10 (ICD 10) and who had never received medication were included in the study. The Montgomery Depression Scale (MADRS) was used to assess depressive symptoms and the Young Mania Rating Scale (YMRS) for manic symptoms. Thyroid function indicators include thyroid-stimulating hormone (TSH), free triiodothyronine (FT3), total triiodothyronine (TT3), free thyroxine (FT4), and total thyroxine (TT4). Levels of TSH, TT4, FT4, TT3, and FT3 were measured within 48 h of hospitalization, between 06:00 and 08:00.

**Results:**

The data analysis finally covered the data of 291 subjects (136 in a bipolar manic group, 128 in a bipolar depressive group, and 27 in a bipolar mixed group), including 140 males and 151 females, with an average age of 27.38 ± 8.01. There was no significant difference in age, sex, marital status, work status, family history, and course of illness among the manic group, depressive group, and mixed group. The level of FT3, the rate of thyroid hormone increased secretion, and the total abnormality rate of thyroid hormone secretion in the manic group were significantly higher than those in the depressive group.

**Conclusion:**

These findings indicate that thyroid functions were significantly different between depressive and manic episodes in BD patients. In clinical practice, it is necessary to take into account the differences in thyroid hormone levels in patients with BD across different emotional states in choosing drug.

## Background

Bipolar disorder (BD) is a chronic episodic illness. It affects more than 1% of the global population [1], with a lifetime prevalence of 0.4–2.4% according to different diagnostic criteria [2]. It has been eating into the vitals of humankind, leading to obesity, loss of work efficiency, impairment of mental functions, deteriorating social relationships, and finally, deliberate self-harm [1, 3–8]. In the World Health Organization (WHO) World Mental Health (WMH) surveys, BD was the second greatest influential of the nine mental disorders and 10 chronic physical disorders surveyed that affected days out of the role [9]. Also, suicide is quite frequent among the population with BD, with the risk of suicide death is up to 10–30 times higher than that of the general population [10].

One of the traits of BD is fluctuations in mood state. The clinical manifestations in patients with BD are extremely diverse, ranging from mild hypomania or mild depression to severe forms of mania or depression accompanied by profound psychotic symptoms. This alternation can occur spontaneously or be triggered by pharmacologic and psychological factors. First, many studies suggest that many types of medications are able to interfere thyroid function, affecting different parts of the functional thyroid circuit, such as lithium [11], quetiapine and olanzapine [12]. Besides, previous studies have shown that different neurotransmitters and neuroactive steroids monoamines, for example melatonin, cortisol, thyroid hormones et.al, may be involved in the pathogenesis of BD, and the hypothalamic-pituitary-thyroid (HPT) axis is the prime candidate [1, 13]. It has been proposed low thyroid function is associated with the depressive phase of BD, due to the antidepressant effect of thyroid hormone in the treatment of bipolar depression [14]. An early report showed that, among patients admitted to hospital due to bipolar depression, those with low baseline TSH levels were more likely to switch to mania [15]. Besides, milder fluctuations in mood in BD patients seem to correlate with the TSH response to TRH stimulation: Increasing severity of mood symptoms seems to be associated with reduced TSH response [16]. Accordingly, changes in thyroid function may cause fluctuations in patients with BD. In other words, thyroid function in BD patients across different emotional states may be different. Further, the thyroid hormone levels can affect the patient’s response to drug therapy. Thyroid dysfunction is associated with poor treatment response in bipolar depression [17]. Therefore, a comprehensive understanding of thyroid function in drug-naive BD patients is essential. The purpose of the present study was to investigate and describe the thyroid function in drug-naive BD patients across different, with the expectation of providing support for treatment options.

## Methods

The current study was a cross-sectional study on thyroid profile, and it was conducted at the Mental Health Center of West China Hospital from December 2018 to December 2019. Thyroid function indicators include thyroid-stimulating hormone (TSH), free triiodothyronine (FT3), total triiodothyronine (TT3), free thyroxine (FT4), and total thyroxine (TT4). All subjects were inpatients and diagnosed independently by two senior doctors, and only patients with the same diagnosis were included in the study. Inclusion criteria:1) ages 18 to 65 years old; 2) a discharge diagnosis with BD according to the International Classification of Diseases diagnostic Criteria, Edition 10(ICD 10)(F31.0-F31.7); 3) drug-naïve before hospitalization. Exclusion criteria included: 1) having comorbid diagnoses of other psychiatric disorders, such as schizophrenia; 2) having serious physical illnesses that needed medications; 3) having evidence of any known autoimmune diseases; 4) pregnant or lactating women; 5) having a history of thyroid disease. All subjects signed informed consent. The China Ethics Committee approved this research of Registering Clinical Trails (ChiECRCT-80,187). And this study completed the clinical trial registration: Chinese Clinical Trials Registry, ChiCTR1800019064, registered 24 October 2018, http://www.chictr.org.cn.

For all subjects, the Montgomery Depression Scale (MADRS) was used to assess depressive symptoms and the Young Mania Rating Scale (YMRS) for manic symptoms. Levels of TSH, TT4, FT4, TT3, and FT3 were measured within 48 h of hospitalization, between 06:00 and 08:00, and blood samples were examined by the laboratory department of west China hospital. Specifically, TSH was detected by electrochemical luminescence double antibody sandwich method, and TT3, FT3, TT4, and FT4 were determined by electrochemical luminescence quantitative analysis (TSH Elecsys cobas e 200, T3 Elecsys cobas e 200, FT3 Elecsys G3 cobas e 200, T4 Elecsys cobas e 200 V2, FT4 Elecsys G2 cobas e 200, all Roche Diagnostics GmbH product). And the stock levels for TSH, FT3, TT3, FT4, and TT4 for healthy subjects, provided by the laboratory department, were 0.27–4.20 mU/L, 3.60–7.50 pmol/L, 1.30–3.10 nmol/L, 12.00–22.00 pmol/L, 62.00–164.00 nmol/L. Increased thyroid hormone secretion (ITHS): at least one of the following indicators is abnormal, decreased TSH (< 0.27 mIU/L), increased TT3 (> 7.50 pmol/L), increased FT3 (> 3.10 nmol/L), increased TT4 (> 22.00 pmol/L), increased FT4 (> 164.00 nmol/L). Decreased thyroid hormone secretion (DTHS): increased TSH (> 4.20 mU/L), decreased TT3 (< 3.60 pmol/L), decreased FT3 (< 1.30 nmol/L), decreased TT4 (< 12.00 pmol/L), decreased FT4 (< 62.00 nmol/L), at least one of the above item abnormal.

All data were analyzed by the statistical package IBM SPSS Statistics software (SPSS). ANOVA or X^2^ tests were performed to test for differences in socio-demographic variables. ANOVA tests was used to compare the hormone levels in different diagnostic subgroups, and X^2^ test for the incidences of abnormal hormone secretion in different diagnostic subgroups. Pearson’s correlation analysis was used to evaluate the correlations among all hormone indicators, demography, total scores of MADRS, total scores of YMRS. All analyses used the two-tailed estimation of significance. All statistically significant *p* values were set at <0.05.

## Results

### Demographic and clinical characteristics data

After applying exclusion criteria, 297 patients with BD were included. In total, 136 people were in the bipolar mania group (BD-M), of whom 16 were hypomanic episodes (BD-M I), 60 were manic episodes without psychotic symptoms (BD-M II), and 60 were manic episodes with psychotic symptoms (BD-M III). There were 128 people in the bipolar depression group (BD-D), among whom 62 were mild to the moderate depressive episode (BD-D I), 43 were major depressive episode without psychotic symptoms (BD-D II), and 23 were major depressive episode with psychotic symptoms (BD-D III). There were 27 patients in the mixed group (BD-MIX) and 6 patients in the remission group (BD-R). The remission group was not included in the data analysis due to its small number.

For the whole study, 291 patients were included in the final data analysis, 140 (48.11%) were male, and 151 (51.89%) were female. 149(51.20%) had a junior high school degree or below, 73(25.09%)had a high school or junior college degree, and 69 (23.71%)had bachelor degree or above. 132(45.36%)were unmarried, 126(43.30%)were married, and 33(11.34%)were divorced or widowed. The mean age was 27.38 ± 8.01 years, the mean age of onset was 20.72 ± 6.26 years, and the illness duration was 6.66 ± 4.98 years. And the mean value of TSH, TT4, FT4, TT3, and FT3 were 2.29 ± 1.47 mU/L, 1.66 ± 1.32 nmol/L, 6.03 ± 10.23 pmol/L, 71.88 ± 39.45 nmol/L, and 13.60 ± 7.23 pmol/L, respectively. The specific demographic and clinical characteristics information of different diagnostic subgroups is shown in Table 1. As show in Table 1, there were no significant difference in age, sex, educational background, marital status, family history and entire disease course among different diagnostic subgroups.

**Table 1:** Demographic and clinical data (mean ± SD) / (n, %)

	The bipolar manic group(BD-M)				The mixed group (BD-MIX)	The bipolar depressive group(BD-D)				total	F/X^2^	p
	BD-M I	BD-M II	BD-M III	total		BD-D I	BD-D II	BD-D III	total			
Number	16	60	60	136	27	62	43	23	128	291	–	–
Age (years)	27.19 ± 10.94	29.71 ± 7.70	26.62 ± 7.77	27.81 ± 8.62	27.41 ± 8.96	25.95 ± 5.71	28.91 ± 8.13	25.78 ± 7.29	26.91 ± 7.04	27.38 ± 8.01	1.366^a^ 0.409^b^	0.241^a^ 0.665^b^
Sex												
male	4 (25.00)	25 (41.67)	28 (46.67)	71 (52.21)	11 (40.74)	28 (45.16)	12 (27.91)	13 (56.52)	53 (41.41)	140 (48.11)	8.208^a^	0.223^a^
female	12 (75.00)	35 (58.33)	32 (53.33)	65 (47.79)	16 (59.26)	34 (54.84)	31 (72.09)	10 (43.48)	75 (58.59)	151 (51.89)	4.563^b^	0.102^b^
Educational												
junior High school and below	8 (50.00)	40 (66.67)	20 (33.33)	68 (50.00)	15 (55.56)	32 (51.61)	23 (34.88)	11 (53.49)	66 (51.56)	149 (51.20)	18.442^a^ 0.413^b^	0.103^a^ 0.981^b^
high school and technical secondary school	5 (31.25)	7 (11.67)	24 (40.00)	36 (26.47)	6 (22.22)	15 (24.19)	9 (39.53)	7 (20.93)	31 (24.22)	73 (25.09)		
bachelor degree or above	3 (18.75)	13 (21.67)	16 (26.67)	32 (23.53)	6 (22.22)	15 (24.19)	11 (25.58)	5 (25.58)	31 (24.22)	69 (23.71)		
Marital status												
unmarried	9 (56.25)	25 (41.67)	32 (53.33)	66 (48.53)	17 (62.96)	22 (35.48)	15 (34.88)	12 (52.17)	49 (38.28)	132 (45.36)	19.284^a^ 6.932^b^	0.082^a^ 0.140^b^
married	6 (37.50)	27 (45.00)	24 (40.00)	57 (41.91)	8 (29.63)	34 (54.84)	22 (51.16)	5 (21.74)	61 (47.66)	126 (43.30)		
divorced or widowed	1 (6.25)	8 (13.33)	4 (6.67)	13 (9.56)	2 (7.41)	6 (9.68)	6 (13.95)	6 (26.09)	18 (14.06)	33 (11.34)		
Employment												
on-the-job or student	14 (87.50)	40 (66.67)	32 (53.33)	86 (63.24)	14 (51.85)	41 (66.13)	32 (74.42)	16 (69.57)	69 (53.91)	169 (58.08)	11.189^a^ 3.390^b^	0.083^a^ 0.184^b^
unemployment (including retirement)	2 (12.50)	20 (33.33)	28 (46.67)	50 (36.76)	13 (48.15)	21 (33.87)	11 (25.58)	7 (30.43)	59 (46.09)	122 (41.92)		
Family history												
no	12 (75.00)	47 (78.33)	51 (85.00)	110 (80.88)	20 (59.26)	48 (77.42)	35 (81.40)	19 (82.61)	102 (79.69)	232 (79.73)	9.925^a^	0.128^a^
yes	4 (25.00)	13 (21.67)	9 (15.00)	26 (19.12)	7 (40.74)	14 (22.58)	8 (18.60)	4 (17.39)	26 (20.31)	59 (22.61)	0.192^b^	0.996^b^
Total disease course (years)	6.44 ± 5.85	6.87 ± 4.81	5.57 ± 4.74	6.42 ± 4.96	6.22 ± 4.93	7.60 ± 4.14	7.30 ± 5.98	5.87 ± 4.63	7.19 ± 4.96	6.66 ± 4.98	1.121^a^ 1.300^b^	0.350^a^ 0.274^b^
MADRS	–	–	–	–	26.54 ± 6.72	23.00 ± 10.70	32.48 ± 9.31	31.11 ± 6.17	25.74 ± 11.79	–	–	–
YMRS	15.75 ± 8.78	29.39 ± 10.00	31.45 ± 12.29	28.57 ± 12.00	21.60 ± 7.62	–	–	–	–	–	–	–
Thyroid profiles												
TSH (mU/L)	2.37 ± 1.14	2.38 ± 1.69	2.21 ± 1.43	2.31 ± 1.51	2.11 ± 1.07	2.30 ± 1.41	2.60 ± 1.87	1.73 ± 0.72	2.30 ± 1.51	2.29 ± 1.47	1.013^a^ 0.200^b^	0.417^a^ 0.819^b^
TT3 (nmol/L)	1.41 ± 0.40	1.62 ± 0.56	2.18 ± 2.74	1.86 ± 1.92	1.53 ± 0.37	1.55 ± 0.40	1.39 ± 0.47	1.59 ± 0.31	1.50 ± 0.40	1.66 ± 1.32	2.043^a^ 2.514^b^	0.060^a^ 0.083^b^
FT3 (pmol/L)	4.51 ± 1.20	9.67 ± 18.25	7.12 ± 12.51	8.10 ± 15.05	4.69 ± 1.12	4.32 ± 1.13	4.00 ± 1.34	4.62 ± 1.05	4.26 ± 1.20	6.03 ± 10.23	2.139^a^ 4.804^b^	0.049^a^ 0.009^b^
TT4 (nmol/L)	64.83 ± 37.16	69.27 ± 53.03	69.07 ± 41.25	68.84 ± 46.50	79.17 ± 34.25	76.82 ± 29.27	66.27 ± 34.08	78.97 ± 37.06	73.66 ± 32.59	71.88 ± 39.45	0.719^a^ 0.965^b^	0.635^a^ 0.382^b^
FT4 (pmol/L)	13.34 ± 5.97	12.92 ± 6.73	13.64 ± 6.32	13.28 ± 6.45	14.07 ± 6.29	14.98 ± 10.27	11.69 ± 6.35	14.19 ± 3.55	13.73 ± 8.27	13.60 ± 7.23	0.998^a^ 0.195^b^	0.427^a^ 0.823^b^

*n*, number, *%* percentage, *SD* standard deviation, *MADRS* Montgomery Depression Scale, *YMRS* Young mania Rating Scale, *TSH* thyroid-stimulating hormone, *TT3* total triiodothyronine, *FT3* free triiodothyronine, *TT4* total thyroxine, *FT4* free thyroxine. ^a^, comparison on 7 different diagnostic subgroups; ^b^, comparison on 3 different diagnostic subgroups combined

### The serum thyroid hormone levels among different diagnostic subgroups

As can be seen from Table 1, TSH, TT3, TT4 and FT4 levels showed no significant differences between the three diagnostic subgroups or the further seven diagnostic subgroups. However, it was found that the FT3 level was significantly different among the 7 diagnostic subgroups. Specifically, FT3: BD-M II > BD-MIX, BD-D I, BD-D II and BD-D III (*p* = 0.037, *p* = 0.004, *p* = 0.006, *p* = 0.045), and there was no significant difference between the remaining groups. Comparing three combined groups, BD-M, BD-MIX, and BD-D, a difference still lay in FT3 levels. BD-M > BD-D (*p* = 0.003), and there was no significant difference between BD-M and BD-MIX (*p* = 0.116) and between BD-D and BD-MIX (*p* = 0.842).

### The incidences of abnormal hormone secretion among different diagnostic subgroups

The abnormality rates of thyroid hormone secretion in different diagnostic subgroups are shown in Table 2. First, comparison was performed on the increase probability of thyroid hormone secretion. Upon comparison, the 7 diagnostic subgroups were different overall (Fisher = 17.595, *p* = 0.002), but after Bonferroni correction, only the increase rate of thyroid hormone secretion of BD-M II was significantly higher than that of BD-D II (20.00% vs 1.61%, pp. < 0.005). Comparing the three combined groups, BD-M, BD-MIX, and BD-D, a difference still lay in the overall distribution of the increase rate of thyroid hormone secretion (20.59, 7.41, 1.56%; X^2^ = 28.469, *p* = 0.000), but after Bonferroni correction, only the increase rate of thyroid hormone secretion of BD-M was significantly higher than that of BD-D (20.59% vs 1.56%, pp. < 0.005). Second, the comparison was performed on the decrease probability of thyroid hormone secretion. No significant difference was found from the comparison among the 7 diagnostic subgroups or among the 3 diagnostic subgroups combined (X^2^ = 6.172, *p* = 0.409; X^2^ = 0.656, *p* = 0.718). At last, comparison is performed on the total abnormality rate (increase + decrease). Upon comparison, the 7 diagnostic subgroups were different overall (X^2^ = 12.822, *p* = 0.045), but after Bonferroni correction, only the abnormality rate of thyroid hormone secretion of BD-M II was found to be significantly higher than that of BD-D III (70.00% vs 30.43%, pp. < 0.005). Comparing the three combined groups, BD-M, BD-MIX, and BD-D, a difference still lay in the overall distribution of the increase rate of thyroid hormone secretion (69.85, 51.85, 45.31%, X^2^ = 15.676, *p* = 0.000), but after Bonferroni correction, only the abnormality rate of thyroid hormone secretion of BD-M was found to be significantly higher than that of BD-D (69.85% vs 45.31%, pp. < 0.005).

**Table 2:** The incidences of abnormal hormone secretion among different diagnostic subgroups (n, %)

	The bipolar manic group(BD-M)				The mixed group (BD-MIX)	The bipolar depressive group(BD-D)				total	X^2^	p
	BD-M I	BD-M II	BD-M III	total		BD-D I	BD-D II	BD-D III	total			
Number	16	60	60	136	27	62	43	23	128	291	–	–
ITHS	0 (0.00)	12 (20.00)	6 (10.00)	28 (20.59)	2 (7.41)	1 (1.61)	1 (2.33)	0 (0.00)	2 (1.56)	32 (11.00)	Fisher = 17.595^a^ 28.469^b^	0.002^a^ 0.000^b^
DTHS	9 (56.25)	30 (50.00)	28 (46.67)	67 (49.26)	12 (44.44)	31 (50.00)	26 (60.47)	7 (30.43)	57 (43.75)	64 (21.99)	6.171^a^ 0.656^b^	0.409^a^ 0.718^b^
Total	9 (56.25)	42 (70.00)	34 (56.67)	95 (69.85)	14 (51.85)	32 (51.61)	27 (62.80)	7 (30.43)	59 (45.31)	96 (32.99)	12.822^a^ 15.676^b^	0.045^a^ 0.000^b^

*n* number; *%* percentage, *ITHS* increased thyroid hormone secretion, *DTHS* decreased thyroid hormone secretion. ^a^, comparison on 7 different diagnostic subgroups; ^b^, comparison on 3 different diagnostic subgroups combined

### Correlative analysis of hormones secretion and symptoms in BD-M, BD-MIX, and BD-D groups

For BD-M patients, pearson’s correlation analysis showed that FT3 was positively correlated with the YMRS total score (*r* = 0.182, *p* = 0.045). But, a correlation coefficient of 0 < |r| ≤ 0.2 is interpreted as almost no correlation. This indicates that the severity of clinical symptoms in BD-M patients is not highly correlated with FT3 levels. For BD-MIX patients, there was no significant correlation found between FT3 levels and the YMRS total score (*r* = − 0.110, *p* = 0.696). And there was no relationship was found between FT3 levels and the MADRS total score (*r* = − 1.58, *p* = 0.120) in the BD-D patients.

## Discussion

The present study finds that, compared with BD-D patients, BD-M patients have higher serum FT3 levels, higher rate of thyroid hormone increased secretion and higher rate of total abnormality thyroid hormone secretion. These findings indicate that thyroid axis dysfunction is more severe in patients with a manic episode than patients with a depressive episode. Differences in age and sex cannot explain this result, as there is no statistically significant difference in age and sex between the BD-M group and the BD-D group. And, this result is not consistent with that obtained by Li C et al., which found no significant differences in thyroid profiles in BD patients with different mood episodes [18]. In addition, the correlation analysis did not find a strong correlation between the severity of clinical symptoms and thyroid hormone levels in patients with BD across different mood states. Previous research has shown that thyroid hormones have a major effect on behavior and even can modulate the phenotypic expression of affective disorders. For example, hyperthyroidism or thyrotoxicosis has been shown to be related to forms of anxiety or mood lability, such as mania [19, 20]. It follows from this, thyroid hormone levels may cause mood changes in BD patients. However, because this study was a cross-sectional study, a causal relationship between changes in thyroid hormone levels and mood swing in patients with BD cannot be demonstrated. Therefore, further prospective studies are needed to verify this hypothesis.

At present, drug therapy is the main treatment for BD. Most psychotropic drugs have thyroid side effects, wherein lithium salt is most well-known. Lithium inhibits the release of thyroid hormones and increases TRH-stimulated TSH, leading to goiter, clinical and subclinical hypothyroidism, but rarely to hyperthyroidism [21–23]. In this study, thyroid function of drug-naïve BD-M patients is active. This indicates that the inhibition of thyroid function by lithium salt may be part of the reason why lithium salt can control acute manic episodes. Atypical antipsychotic drugs can, according to their dopamine antagonism profile, moderately interfere with TSH response to TRH without provoking major disturbances to thyroid function [12]. A recent population-based naturalistic study indicated that, lower serum FT4 levels were associated with the use of antipsychotics (*p* = 0.001), particularly quetiapine (*p* = 0.003) and olanzapine (*p* = 0.018), while there were no associations with serum TSH levels [24]. Reductions in TT4 and FT4 are the most common change in thyroid hormones with antidepressant treatment [25, 26]. Besides, compared with the use of antipsychotics alone, using antipsychotics in combination with other psychotropic drugs, especially with antidepressants, was associated with lower FT4 level (*p* < 0.001, 24]. Therefore, the side effects of psychotropic drugs may be beneficial for BD-M patients, but not for BD-D patients. In addition, in this study, 43.75% of untreated patients with bipolar depression had decreased thyroid hormone secretion. It is necessary to avoid the use of drugs that inhibit thyroid function, which may aggravate depression symptoms and reduce the effect of antidepressant treatment, in patients with bipolar depression [17]. It has been reported that, in rapid-cycling bipolar disorder and refractory bipolar disorder, adjunctive thyroid hormones can be effective [27–30]. Although findings from studies of the efficacy of thyroid augmentation have not been uniformly positive [31], in general, these strategies remain alternatives for treatment-resistant non-bipolar and bipolar depressions [32, 33]. In clinical practice, therefore, it is necessary to take into account the differences in thyroid hormone levels in patients with BD across different emotional states in choosing drug.

To our knowledge, this is the first study to independently compare thyroid hormone levels in different diagnostic subgroups of patients with drug-naive BD. One major advantage of the present study compared to previous studies was that all subjects were drug-naïve, thus avoiding the impact of drugs on thyroid function.

The current study has serval limitations. First, hormonal variables were evaluated at a single time point. Because TSH, T3, FT3, T4, and FT4 tests can vary significantly between day and night, the acquisition of hormone levels at multiple time points is an ideal choice to establish the reliability of hormone levels. Besides, we did not check thyroid auto antibodies, and could not rule out underlying thyroid disease. Second, compared with the BD-M and BD-D groups, people in the BD-MIX group were too few, which may cause errors, so that cautions should be remained about the result that there was no significant difference in thyroid function between BD-M group and BD-D group and BD-MIX group. And, it have been found long that thyroid axis dysfunction is more common in bipolar mixed than in bipolar manic patients [34]. Finally, this study is a cross-sectional study and cannot prove whether mood fluctuations in bipolar disorder are related to changes in thyroid function. Further prospective research is required.

## Conclusion

These findings indicate that thyroid functions were significantly different between depressive and manic episodes in BD patients. In clinical practice, it is necessary to take into account the differences in thyroid hormone levels in patients with BD across different emotional states in choosing drug.

## Data Availability

The datasets used and/or analysed during the current study are available from the corresponding author on reasonable request.

## Abbreviations

BD: Bipolar disorder
ICD 10: International Classification of Diseases diagnostic Criteria, Edition 10 who
MADRS: Montgomery Depression Scale
YMRS: Young Mania Rating Scale
TRH: Thyrotropin-releasing hormone
TSH: Thyroid-stimulating hormone
FT3: Free triiodothyronine
TT3: Total triiodothyronine
FT4: Free thyroxine
TT4: Total thyroxine
